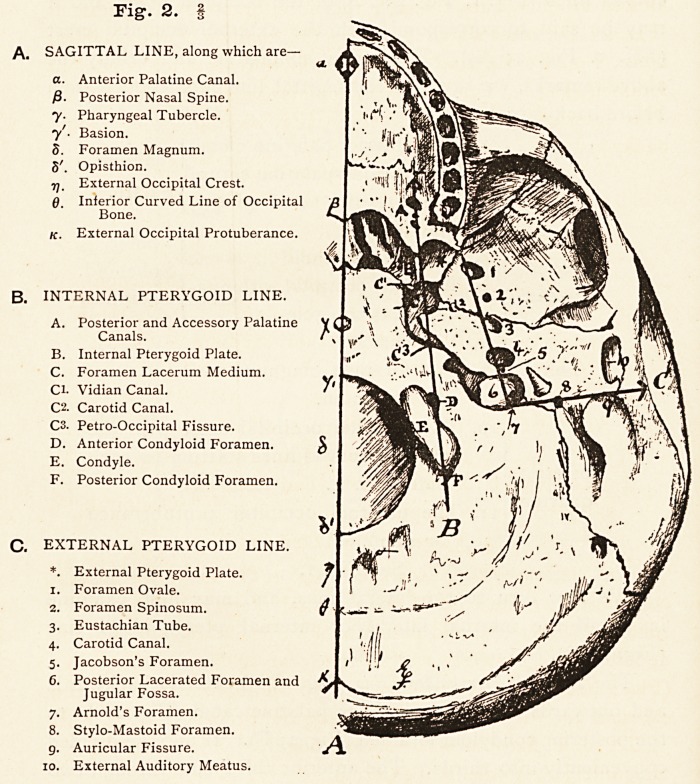# On the Localisation of the Foramina at the Base of the Skull

**Published:** 1895-09

**Authors:** Edward Fawcett

**Affiliations:** Professor of Anatomy in University College, Bristol


### ON THE LOCALISATION OF THE FORAMINA AT THE BASE OF THE SKULL.

BY

EDWARD FAWCETT, M.B., C.M. Edin.,
*Professor of Anatomy in University College, Bristol.*

---

IF I mistake not, the foramina at the base of the skull have at all times proved more or less of a bugbear to students of medicine; and there are, perhaps, many reasons why they should, and should have done so; for with the exception of the foramen magnum they are comparatively small in size, and they are, moreover, crowded into a small space. It is not possible for a student to see them properly at the distance allotted him in the lecture theatre, nor, unless the class be a very small one, is it always possible for the teacher to demonstrate each foramen to every member of the class. To a certain extent he gets over the difficulty—if he be a good draughtsman—by drawing a diagram. If not a good draughtsman, he perhaps has recourse to some diagram specially drawn for him. If not the possessor of that, he delivers his lecture, passing during the time a whalebone bristle through various foramina, and by the time he has finished has probably left his audience in a complete muddle, though not without inspiring its members with a feeling of respect—even awe—for the base of the skull. He may, perchance, have succeeded in instilling into the mind of at least one student the idea that the œsophagus passes through the foramen magnum! Little wonder is there that the student who passes his elementary examination on osteology after having the "hard luck" to be examined on the base of the skull, is looked up to by his fellow students as being something out of the common; little wonder that he has more respect for himself. But, the same man who is able to recognise these foramina when he sees them may be utterly at sea when asked to write down their relative positions with even tolerable accuracy.

If it be a difficult matter for the student in harness, how much more difficult must it be for the almost unavoidably rusty practitioner who, through the circumstances of practice, is unable to keep himself in touch with all his former studies!

To meet these difficulties is the object of this paper. The methods described in it have for some time been used by me in teaching my own students, with the most gratifying success; and it is my desire that they may meet with the same success whenever, wherever, and by whomsoever they are used.

If we examine the inferior aspect of the base of the skull we are at once struck with the number of foramina in it, and, moreover, if we compare it with a plate such as is exhibited in any book on Osteology with a view to learning the names and positions of these foramina, we soon find that we have set ourselves a formidable task. But the burden of that task is very much lightened if it occur to us to systematise the work. That work is rendered less irksome when it is pointed out to us that the foramina and, in addition, the main objects of interest at the base resolve themselves into antero-posterior rows. Many of these, for instance, lie in the middle line, along a line drawn from the interval between the central incisor teeth in front and the external occipital protuberance behind.

Many lie in a line drawn backwards and outwards between the posterior palatine foramina and the posterior condyloid foramen.

Many lie along a line drawn from the base of the external pterygoid plate of the sphenoid to the middle of the posterior wall of the posterior lacerated foramen, and thence continued outwards to the interval between the mastoid process and the external auditory meatus.

As the skull is for all practical purposes bilaterally symmetrical, it follows that these lateral rows of foramina, etc., are present on both sides of the middle line, so that the main objects at the base of the skull as seen from the exterior resolve themselves into five rows—namely, one median and therefore single row, and two pairs of lateral rows.

It is convenient to call the line running through the median row of objects, the *Sagittal Line:* concerning the two lateral lines it is convenient to speak of the internal as the *Internal*

*Pterygoidean Line*, because it commences just in front of the internal pterygoid plate of the sphenoid and soon runs through it; the external lateral line commences at the base of the external pterygoid plate of the sphenoid, and may on that account be termed the *External Pterygoidean Line*. These latter lines are paired. We have thus located five antero-posterior lines on the base of the skull, two of which are paired, which reduces the number to three, viz.:—

| | |
|---|---|
| 1. Median or Sagittal (1). | Fig. 2 A. |
| 2. Internal Pterygoidean (2). | Fig. 2 B. |
| 3. External Pterygoidean (2). | Fig. 2 C. |

Of course these lines are all imaginary ones. Let us now prove the value of them, beginning with the sagittal line.

The *Sagittal Line* (Fig. 1, and Fig. 2; A), as has been said before, is drawn backwards from the interval between the central incisor teeth to the external occipital protuberance.

It can with great advantage be divided into fourths, the anterior or first fourth lies on the hard palate reaching behind to the tip of the posterior nasal spine (Fig. 2; $\beta$); the second fourth bridges the interval between the posterior nasal spine and the middle of the anterior wall of the foramen magnum; the third fourth bridges the foramen magnum itself, and the posterior fourth extends from the middle of the posterior wall of that foramen to the external occipital protuberance (Fig. 2; $\kappa$).

The anterior fourth may be roughly divided into three parts, and just in front of the junction of its anterior and middle thirds we find the anterior palatine canal (Fig. 2; A$a$), which, as is well known, is made up of four parts—two lateral, the canals of Stenson, and two antero-posterior, the foramina of Scarpa. At the junction of the middle and posterior thirds we find the maxillo-palatine suture, and at the posterior end of this hindmost third is the posterior nasal spine (Fig. 2; A$\beta$).

The anterior fourth of this sagittal line is thus seen to cut through from before backwards, the anterior palatine canal, the maxillo-palatine suture, and the posterior nasal spine.

The second fourth may also be divided into thirds, but in doing so we must look vertically over it. When we do this we see that the anterior third covers the vomerine part of the nasal septum the middle third the basi-sphenoid and the posterior third the basi-occipital, and we see besides that the posterior third cuts through the pharyngeal tubercle (Fig. 1.

**Fig. 1.**

MEDIAN or SAGITTAL LINE. $\frac{2}{3}$

Fig. 2; A$\gamma$) of the occipital bone about half an inch in front of the foramen magnum.

The third fourth bridges the foramen magnum (Fig. 1. Fig. 2; A$\delta$), dividing it into two halves and connects the basion (Fig.

1. Fig. 2; $A\gamma^{1}$) in front with the opisthion (Fig. 1. Fig. 2; $A\delta^{1}$) behind. The hindmost fourth extending between the middle of the posterior wall of the foramen magnum (opisthion) in front, and the external occipital protuberance (inion) (Fig. 1. Fig. 2; $A\kappa$), behind is bisected by the emergence from it of the inferior curved lines (Fig. 1. Fig. 2; $A\theta$) of the occipital bone, and it may be said to correspond with the external occipital crest (Fig. 1. Fig. 2; $A\eta$). If we now summarise and tablify the above remarks, we say that the sagittal line cuts through from before backwards.

| | | |
|---|---|---|
| Anterior quarter | 1. The anterior palatine canal | Maxilla. |
| | 2. The maxillo-palatine suture | |
| | 3. The posterior nasal spine ... | Palate. |
| Second quarter | 4. The vomer ... ... ... | Vomer. |
| | 5. The basi-sphenoid ... ... | Sphenoid. |
| | 6. The basi-occipital with pharyngeal tubercle ... ... | Occipital. |
| | 7. The basion. | |
| Third quarter | 8. The foramen magnum. | |
| | 9. The opisthion. | |
| Posterior quarter | 10. The external occipital crest with the inferior curved lines starting on each side from it. | |
| | 11. The external occipital protuberance or inion. | |

See Fig. 1.

We now turn to the lateral lines, and may first examine in detail the internal lateral or internal pterygoidean line (Fig. 2; B).

The *Internal Pterygoidean line* (Fig. 2; B) extends backwards and outwards from the posterior palatine canal (Fig. 2; A) to the posterior condyloid foramen (Fig. 2; F). It may be divided conveniently into thirds. The anterior third cuts through two bones, the palate and the sphenoid (internal pterygoid plate). The middle third cuts through the petrous part of the temporal bone, and the posterior third cuts through part of the occipital bone.

The anterior third commences in the posterior palatine canal, soon cuts through the accessory palatine canals (Fig. 2; A), and then the internal pterygoid plate of the sphenoid (Fig. 2; B) with the base of the hamular process; at the root of the pterygoid plate it cuts through the pterygoid tubercle, under cover of which

**Fig. 2.** $\frac{2}{3}$

**A.** SAGITTAL LINE, along which are—

- α. Anterior Palatine Canal.

- β. Posterior Nasal Spine.

- γ. Pharyngeal Tubercle.

- γ′. Basion.

- δ. Foramen Magnum.

- δ′. Opisthion.

- η. External Occipital Crest.

- θ. Inferior Curved Line of Occipital Bone.

- κ. External Occipital Protuberance.

**B.** INTERNAL PTERYGOID LINE.

- A. Posterior and Accessory Palatine Canals.
- B. Internal Pterygoid Plate.
- C. Foramen Lacerum Medium.
- $C^1$. Vidian Canal.
- $C^2$. Carotid Canal.
- $C^3$. Petro-Occipital Fissure.
- D. Anterior Condyloid Foramen.
- E. Condyle.
- F. Posterior Condyloid Foramen.

**C.** EXTERNAL PTERYGOID LINE.

- *. External Pterygoid Plate.
- 1. Foramen Ovale.
- 2. Foramen Spinosum.
- 3. Eustachian Tube.
- 4. Carotid Canal.
- 5. Jacobson's Foramen.
- 6. Posterior Lacerated Foramen and Jugular Fossa.
- 7. Arnold's Foramen.
- 8. Stylo-Mastoid Foramen.
- 9. Auricular Fissure.
- 10. External Auditory Meatus.

is the posterior end of the Vidian canal (Fig. 2; $C^1$), and terminates in the large, jagged foramen lacerum medium (Fig. 2; C).

The middle third commencing in this middle lacerated foramen cuts almost immediately on its way backwards the upper end of the carotid canal (Fig. 2; $C^2$) which opens into the outer wall of the foramen lacerum medium; behind that, at a distance of half an inch it crosses the petro-occipital fissure (Fig. 2; $C^3$), when the posterior third takes up the running.

The posterior third, commencing in the petro-occipital suture, almost at once cuts through the anterior condyloid foramen (Fig. 2; D), after which it crosses the condyle of the occipital bone (Fig. 2; E), and terminates in the posterior condyloid foramen (Fig. 2; F). We may summarise and tablify thus:—The internal pterygoidean line cuts through from before backwards.

| | | |
|---|---|---|
| Anterior Third | 1. Posterior palatine canal ... | Palate. |
| | 2. Accessory palatine canals ... | |
| | 3. Hamular process ... ... | Sphenoid. |
| | 4. Internal pterygoid plate ... | |
| | 5. Pterygoid tubercle ... ... | |
| | 6. Vidian canal ... ... | |
| | 7. Foramen lacerum medium | Temporal. |
| Middle Third | 8. Carotid canal (upper and inner opening) ... ... | |
| | 9. Petrous—temporal ... ... | |
| Posterior Third. | 10. Petro-occipital suture ... | Occipital. |
| | 11. Anterior condyloid foramen | |
| | 12. Occipital condyle ... ... | |
| | 13. Posterior condyloid foramen | |

The *External Pterygoidean line* (Fig. 2, C), as we have seen, commences at the base of the external pterygoid plate of the sphenoid (Fig. 2; *), and passes backwards and outwards to the middle of the posterior wall of the foramen lacerum posterius (Fig. 2; 6), when it turns outwards to the interval between the external auditory meatus and the mastoid process (Fig. 2; 9).

This line, like the last, may be roughly divided into thirds; viz., anterior, middle, and external.

The anterior third, commencing at the root of the external pterygoid plate of the sphenoid, at once cuts through the inner side of the foramen ovale (Fig. 2; 1), then the foramen spinosum (Fig. 2; 2), then the spine of the sphenoid, behind which it ends in the osseous Eustachian tube (Fig. 2; 3), having covered the great wing of the sphenoid bone. The middle third, commencing at this Eustachian tube, crosses the petrous temporal, cutting through immediately behind the Eustachian tube the outer and lower opening of the carotid canal (Fig. 2 ; 4), then a plate of bone separating that canal from the jugular fossa and the posterior lacerated foramen, and in that plate it cuts through the foramen through which Jacobson's nerve emerges (Fig. 2; 5), (Jacobson's foramen), and terminates in the posterior wall of the posterior lacerated foramen (Fig. 2 ; 6), whose inner bay transmits the inferior petrosal sinus, middle bay transmits the 9th, 10th, and 11th cranial nerves, and whose outer bay transmits the lateral sinus.

The external third, commencing at the middle of the posterior wall of the posterior lacerated foramen, and terminating between the mastoid process and the external auditory meatus, follows the line of the petro-mastoid suture. It cuts through the jugular fossa (Fig. 2 ; 6), and in its outer wall the foramen for Arnold's nerve (Fig. 2; 7), after which it cuts through the stylo-mastoid foramen (Fig. 2 ; 8), and lastly the auricular fissure (Fig. 2 ; 9). The external pterygoidean line thus cuts through on its way backwards and upwards :

| | | |
|---|---|---|
| Anterior Third | 1. The base of the external pterygoid plate ... ... ... ...<br>2. The foramen ovale ... ... ...<br>3. The foramen spinosum ... ...<br>4. The spine of the sphenoid... ... | Sphenoid. |
| Middle Third | 5. The Eustachian tube and canal for tensor tympani muscle ... ...<br>6. The carotid canal (lower and outer opening) ... ... ... ...<br>7. Plate of bone between carotid canal and posterior lacerated foramen, containing Jacobson's foramen... | Temporal. |
| External Third | 8. The posterior lacerated foramen.<br>9. The jugular fossa.<br>10. The stylo-mastoid foramen.<br>11. The auricular fissure. | |

In all cases these lines are drawn without regard to the contour of the base of the skull. It is essential to keep this in mind. Thin wires stretched between the rallying points answer well. The median line is six inches long on the average; the internal pterygoidean is three inches long; and the external pterygoidean in its anterior two-thirds is two inches, in its external one-third one inch long. These are, of course, average measurements.

## Figures and Tables

**Fig. 1. f1:**
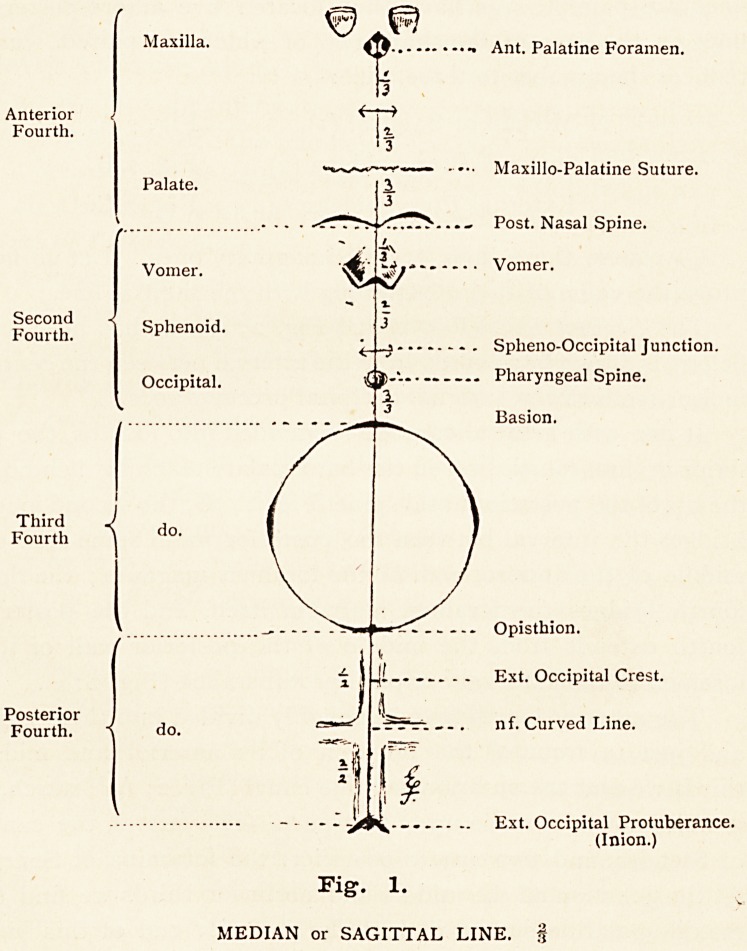


**Fig. 2. f2:**